# Toward Regeneration of the Heart: Bioengineering Strategies for Immunomodulation

**DOI:** 10.3389/fcvm.2019.00026

**Published:** 2019-03-21

**Authors:** Arianna Ferrini, Molly M. Stevens, Susanne Sattler, Nadia Rosenthal

**Affiliations:** ^1^Department of Materials, Imperial College London, London, United Kingdom; ^2^National Heart and Lung Institute and BHF Centre for Research Excellence, Imperial College London, London, United Kingdom; ^3^Department of Bioengineering, Imperial College London, London, United Kingdom; ^4^Institute of Biomedical Engineering, Imperial College London, London, United Kingdom; ^5^The Jackson Laboratory, Bar Harbor, ME, United States

**Keywords:** myocardial infarction, injectable hydrogel, cardiac regeneration, immunomodulation, growth factors

## Abstract

Myocardial Infarction (MI) is the most common cardiovascular disease. An average-sized MI causes the loss of up to 1 billion cardiomyocytes and the adult heart lacks the capacity to replace them. Although post-MI treatment has dramatically improved survival rates over the last few decades, more than 20% of patients affected by MI will subsequently develop heart failure (HF), an incurable condition where the contracting myocardium is transformed into an akinetic, fibrotic scar, unable to meet the body's need for blood supply. Excessive inflammation and persistent immune auto-reactivity have been suggested to contribute to post-MI tissue damage and exacerbate HF development. Two newly emerging fields of biomedical research, immunomodulatory therapies and cardiac bioengineering, provide potential options to target the causative mechanisms underlying HF development. Combining these two fields to develop biomaterials for delivery of immunomodulatory bioactive molecules holds great promise for HF therapy. Specifically, minimally invasive delivery of injectable hydrogels, loaded with bioactive factors with angiogenic, proliferative, anti-apoptotic and immunomodulatory functions, is a promising route for influencing the cascade of immune events post-MI, preventing adverse left ventricular remodeling, and offering protection from early inflammation to fibrosis. Here we provide an updated overview on the main injectable hydrogel systems and bioactive factors that have been tested in animal models with promising results and discuss the challenges to be addressed for accelerating the development of these novel therapeutic strategies.

## Immune Features of Cardiovascular Disease and Heart Failure

Cardiovascular diseases (CVDs) is the leading cause of death worldwide (1). In Europe only, CVDs claim more than 4 million lives each year, accounting for 49% of death among women and 40% among men (2). Myocardial infarction (MI) is the most common CVD (1). MI occurs when the supply of freshly oxygenated blood to the left ventricle is blocked as a result of a prolonged coronary artery occlusion. This occlusion and the resulting ischemic damage cause the loss of up to 1 billion cardiomyocytes (CMs) (3). Given the limited intrinsic regenerative capacity of the heart, surviving cardiomyocytes increase in size more than in number (4) and a non-contractile fibrous scar is formed to avoid fatal cardiac rupture.

Although cardiomyocyte death initiates an inflammatory response that is essential for early removal of necrotic debris and scar formation, persistent inflammation can cause immune-mediated tissue damage (5). Anti-cardiac autoreactivity of the adaptive immune system progressively contributes to structural remodeling and functional decline (6–8), inducing morphological changes such as left ventricular (LV) remodeling that compromises the function and pumping capacity of the heart. These cumulative immune responses lead to the progressive spiral toward heart failure (HF) (9), where the heart is incapable of generating sufficient cardiac output to meet the demands of the body.

HF has a poor prognosis and no effective treatment is available to date (10), the disease is associated with poor quality of life, high healthcare costs, and a high mortality rate (11, 12). Current treatments comprise pharmacotherapy, medical devices such as ventricular assist devices, and heart transplant. The first two strategies optimize the function of the remaining viable cardiomyocytes, and although successful in saving and improving quality of life they do not address the underlying causes of cell loss and restore lost cardiac tissue, and therefore cannot effectively manage the progression to HF. Heart transplantation remains the only effective treatment to restore fully functioning cardiac tissue (13, 14), but the limited amount of available donors and complications from immune rejection make it impractical for the number of people affected by HF (15). As a result, there is a pressing need for effective methods to regenerate damaged myocardium and prevent adverse remodeling.

Here we discuss the status quo of tissue engineering approaches to improve cardiac regeneration after MI, and how intended or fortuitous immunological properties of biomaterials may underlie their beneficial effects. We first focus on injectable *in situ* gelling systems and the relative material design criteria that need to be considered for cardiac repair applications. We then present an overview of the main bioactive molecules with pro-angiogenic, anti-apoptotic and anti-inflammatory properties, delivered *in vivo* as single or combined factors in animal models of MI. Finally, we discuss some of the challenges when considering a clinical translation of these strategies.

## Hydrogels As a Novel Therapeutic Approach After MI

Tissue engineering and regenerative medicine have recently emerged as a prospective option for MI and HF treatment. The availability of engineered or regenerated cardiac tissue to supplant donated hearts would be a significant step forward in improving patient prognosis and advance treatments for MI. Biomaterial-based strategies are being developed to tackle cardiac regeneration post-MI, strengthened by the conclusion that 95–99% of therapeutic stem cells delivered to the infarcted heart are lost within the first 24 h (16–18). In addition to providing mechanical support for the damaged myocardial wall, tissue engineering strategies have the potential to enhance cell survival and cell retention, reducing immediate cell loss due to mechanical washout and promoting integration into the host tissue. Biomaterials not only to address the issue of cell engraftment but also aid in the clinical translation of therapies based on bioactive molecules, systemic administration of which is hindered by their short *in vivo* half-life, the need for repeated injections and high cost of treatment. By stably delivering bioactive molecules to the area of damage, biomaterials have the potential to counteract the morphological changes leading to HF by promoting angiogenesis and cell survival.

The two main strategies currently implemented for cardiac tissue engineering are cardiac patches or scaffolds and *in situ* gelling systems. Engineered scaffolds or patches are solid porous polymeric matrices which may have cells and/or bioactive molecules attached to them (19). These approaches are limited by the invasive procedure by which they are applied, as they require invasive surgeries for implantation and is not an option for patients with advanced HF, due to comorbidities that exclude them from undergoing surgery. In the 1990s, a therapeutic strategy involved ventricular restraints such as polymeric meshes enfolding the heart or sutured to its surface. Several studies have shown that they are effective in reducing infarct expansion by mechanically stabilizing the heart, limiting long-term changes in the LV geometry in large animal models (20–22) with minimal options for clinical translation (23), leaving cardiac patches and *in situ* gelling systems as the two main approaches currently investigated for cardiac tissue engineering (24).

In the gel category, hydrogels have received considerable attention as “water-swollen polymer networks” (23) that have a high percentage of water content similar to human tissue (25). They can be prepared from polymers of either natural or synthetic origin and they are able to absorb a considerable amount of water or biofluids, resulting in swelling with maintenance of their shape (26). As injectable fluid, they represent a minimally invasive approach (27) used for the localized delivery of bioactive molecules to target sites, allowing for well-controlled release kinetics and increasing the functional half-life of cargo molecules. As a delivery vehicle, hydrogels can also be used for “combination strategies,” simultaneously encapsulating cells and bioactive molecules.

## Design of Injectable Biomaterials for Cardiac Tissue Engineering

When designing any biomaterial for tissue engineering application, important considerations include the function and composition of the target tissue. Biomaterials should have biological and physical properties mimicking those of the target tissue and should be able to degrade upon tissue regeneration. Materials for myocardial tissue engineering should ideally be designed to offer mechanical support to the infarcted myocardium, while not interfering with synchronization and myocardial geometry. In this respect, an injectable/*in situ* polymerizable material is advantageous over a patch because upon injection it can uniformly disperse in the myocardial wall remaining anchored by interstitial placement (28).

### Injectability and Delivery Methods

A hydrogel that can pass through a fine gauge needle (27G) can be safely administered through intramyocardial injection with a minimally invasive approach (29). Injectability is achieved when the gelation of the hydrogel is either initiated but not completed in the needle or initiated and completed after delivery at the target site. Importantly, polymerization time should be in the order of tens of minutes or shorter so that to ensure the hydrogel is successfully localized and not washed out with the contraction of the heart (30). In the context of cardiac repair, injectable hydrogels represent a minimally invasive approach, decreasing the damage incurred to the targeted and surrounding tissue during delivery (27). The ideal method of delivery providing a quick path to clinical translation would use current catheter technology, but although highly patient-compliant, it poses some challenges for the designing the material which must be maintained in liquid form for the duration of the injection procedure (up to 1 h) and then solidify into a hydrogel once within the myocardial wall. A thermo-responsive hydrogel delivered through a cooled injection system could overcome this challenge.

There are three main methods for the clinical delivery of injectable hydrogel therapies, each with its advantages and limitations. The first is intracoronary injection with a catheter via an inflated percutaneous transluminal coronary angioplasty (PTCA) balloon. This approach is unique as it exploits leaky vessels in the damaged region for the delivery, instead of a puncture injection, and it utilizes currently available technology without the need for additional training. However, it requires special material design and the volume of delivered material cannot be controlled well due to passive leakage from the target vessel (27). A second approach is direct epicardial injection into the heart; advantages of this method include an accurate location control while a drawback is that endoscopic application or open chest access required for the administration are invasive procedures. Lastly, another less invasive approach is trans-endocardial injection via catheter combined with imagining technology such as NOGA® (31). However, this method requires specialized training for both the injection catheter and the imaging modality so is not currently routinely performed (27).

### Stiffness and Mechanical Support

Considerations regarding the mechanical requirements of a biomaterial must take into account the *in vivo* model where it will be tested, since the forces exerted by a human heart will vastly differ from those of a small rodent. Physiologically, the stiffness of the human myocardium ranges from 20 kPa at end-diastole to 500 kPa at the end-systole (32, 33), whereas that of the rat myocardium goes from 0.1 to 140 kPa (33). As the collagenous scar forms, the infarcted myocardium undergoes a time-dependent stiffness change becoming less and less flexible. As measured by atomic force microscopy (AFM), the baseline elastic modulus, which measures the ability of a material to resist deformation, of non-infarcted rat myocardium is 18 ± 2 kPa (34). In the first 24 h after MI the stiffness of infarcted myocardium is relatively soft (4–17 kPa) (35), whereas in the 2 weeks post-MI, significant fibrosis is formed resulting in a threefold increase in the elastic modulus (55 ± 15 kPa) (34). It is known that the physical characteristics of the microenvironment have an effect on the survival and differentiation of the engrafted cells (35). Therefore, the response to cell and material implantation will vary with the stiffness of the infarcted tissue, which in turn depends on the time post-infarction (30). A material envisioned to provide long-term mechanical support should have a high-end stiffness, matching the one of the native myocardium, whereas a material designed for the injection and the delivery of cells and/or bioactive factors should have a low stiffness, as long as it is able to withstand the contraction/dilation of the heart.

### Application Time Post-MI

Since recent and old infarcts have their distinct features and challenges, an important consideration for material design is the time post-MI at which the material is injected. Depending on injury stage and extent of remodeling, the status of MI is categorized as acute (1–7 days), sub-acute (1–3 weeks) or chronic (>1 month). Application of therapeutic agents in chronic models has so far shown little success in improving regeneration, and regenerative therapy in chronic MI remains a challenge. While acute MI involves early ventricular damage, chronic MI changes the ventricular shape, causing a time-dependent dilation, and LV hypertrophy (36). Delivering therapeutic factors shortly after an MI might protect the remote myocardium, minimize scar formation and ultimately attenuate pathological remodeling. However, delivering cells or bioactive molecules too soon risks exposing them to a hostile ischemic and inflammatory environment. The time of injection in *in vivo* studies ranges from immediate (37–40) to 1 week (41, 42) to 2 months post-MI (37). The most common approach in animal models is an immediate injection, which unfortunately does not accurately mimic the clinical situation because patients would not receive a therapy for a minimum of several hours, if not days, post-MI. Hence, studies investigating the effect of biomaterial injections at later time points such as 2 months are valuable to test the possibility to avoid scar expansion and LV dilation in patients that did not receive an earlier treatment (27). Many studies have looked at the importance of application timing, showing how it underpins the benefit seen in the long term. For example, in a murine model of MI, intramyocardial injection of a collagen matrix is only effective at preventing negative ventricular remodeling and long-term decline of cardiac function if administered 3 h after MI rather than 1 week or 2 weeks after ^[^38]. On the other hand, a study investigating the effect of an injectable alginate implant showed beneficial effects both in recent (<7 days) and old (60 days) infarctions, indicating how other concomitant factors (animal model used, one vs. multiple injections, sites of the injections) could contribute to the observed outcome (37).

**Figure 1:**
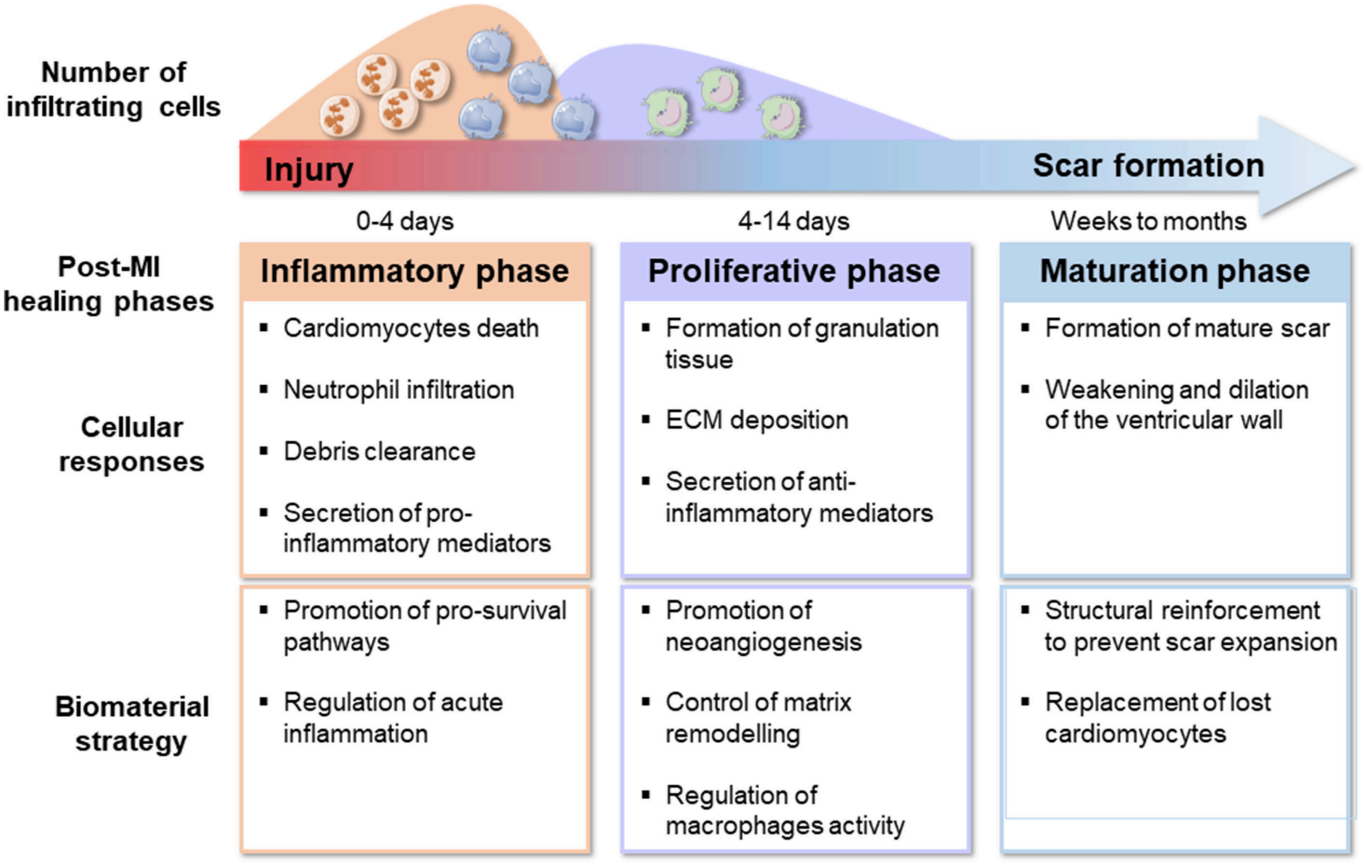
Representation of biomaterial strategies to tackle different post-MI phases.

Timing of therapy may be particularly crucial if an immunomodulatory component is included. The immune response after an MI follows a time-dependent course with distinct but overlapping phases and the outcome of a specific immunomodulatory treatment will be highly dependent on the prevailing inflammatory environment (43), as depicted in Figure 1. During the early inflammatory phase, pro-inflammatory monocytes and macrophages together with neutrophils infiltrate the injured area, clearing the necrotic cardiomyocytes (44). Here, the target for a biomaterial-based strategy would be to promote cardiomyocyte survival and attenuate leukocytes and acute inflammation by supporting efficient resolution of inflammation. The second, proliferative, phase is characterized by fibroblasts and endothelial cell proliferation, ECM deposition and formation a vascularized granulation tissue, accompanied by recruitment of lymphocytes, angiogenesis, and myofibroblasts differentiation (44). At this stage, a biomaterial therapy should aim at regulating macrophage phenotype and function, controlling fibroblast activity and matrix remodeling and promoting angiogenesis. During the final maturation phase, myofibroblast activation recedes and a mature collagen-rich scar is formed which is mostly devoid of cardiomyocytes, hence non-contractile (44). At this point, the goal for any strategy is the replacement of lost cardiomyocytes, potentially delivering cells through a biomaterial approach. In parallel, immunomodulatory strategies may need to forego local factor delivery and aim at targeting ongoing systemic immune activation (8).

### Responsiveness to External Stimuli

An advantage of using injectable delivery systems is the opportunity to tailor polymerization and drug release characteristics to respond to clinically relevant stimuli present in the target tissue microenvironment. Stimulus-responsive “smart” polymers (45) have the unique property of undergoing gelation or cargo-release in response to environmental stimuli (46). Stimuli that have been exploited for regenerative medicine applications *in vitro* and *in vivo*, including cardiac repair, include temperature (47, 48), pH (49, 50), light (28), and enzymes such as metalloproteinases (51).

Temperature-responsive and biodegradable hydrogels are usually in the form of an injectable solution at room temperature, becoming a biodegradable solid at 37°C. These are advantageous for cardiac repair since they undergo a gelation process under physiological conditions that allow them to support cell viability and bioactivity of the encapsulated molecules while avoiding damage to the adjacent tissue (52). Another stimulus that can be used to trigger hydrogel polymerization is pH, particularly relevant in the context of myocardial infarction where the pH drops from the physiological value of 7.4 to 6.5–6.8 due to the switch from aerobic to anaerobic metabolism (53). An acidic microenvironment such as the ischemic myocardium is also an endogenous danger signal alerting the innate immune system and contributing to inflammation (54). Specifically, the acidification of the environment cause macrophages to increase the secretion of the pro-inflammatory cytokines IL-1β and IL-18 (54). Since IL-1 antagonist therapies are being developed for the treatment of MI (55–57), the use of a pH-sensitive drug delivery hydrogel system in a context where IL-1 levels are elevated would potentially be an effective and novel anti-inflammatory therapeutic strategy.

### Bioactivity of the Released Molecules

Another important parameter to consider when designing injectable hydrogels is their ability to protect bioactivity of their biological cargo. In this respect, studies testing these systems *in vivo* should compare the bioactivity of the native factor with the released factor. In general, growth factors and cytokines are unstable molecules with short *in vivo* half-lives. In the inflammatory and acidic environment of the infarcted myocardium, the bioactivity and stability of these molecules is further decreased due to the abundance of proteases and high oxidative stress (58). Hence, hydrogels can act as reservoirs for the sustained and localized delivery of these important regulators under protease-rich conditions. Pioneering work by Langer and Folkman showed that molecules encapsulated in polymer matrixes can display a sustained released for up to 100 days (59). Usually, molecules released from hydrogels exhibit an initial “burst release,” followed by a sustained release profile with slower release rate; however, release kinetics are unique to every biomaterial-cargo combination and must be determined in each case.

A novel class of injectable hydrogels that present a promising option in regard to maintain the bioactivity of the cargo are coacervate hydrogels (60). They are formed by polyelectrolytes of opposing charges mixed together to form droplets held by electrostatic forces (61). Once encapsulated within the coacervate phase, unstable proteins or drugs are separated and protected from the surrounding environment, thereby preserving their bioactivity (60). Moreover, due to their size, coacervates can be applied via very fine gauge needles, important for minimizing damage associated with injection (60).

### Biocompatibility and Immunomodulation

Biocompatibility is defined as “the ability of a material to perform with an appropriate host response in a specific application” (62). The concept of biocompatibility is a characteristic of the material-host interaction more than a material property (63). Specifically, the material should not initiate a substantial foreign body response *in vivo*. This does not preclude the activation of an immune response but shifts the focus on controlling the type and the magnitude of the response in order to prevent further cardiac damage (29). In the past, emphasis was put on minimizing the inflammatory response to biomaterials altogether. However, recent studies demonstrate that the endogenous healing and regeneration processes can be supported by fine-tuning the balance between pro- and anti-inflammatory response (64). As a relevant example, macrophages are highly dynamic and responsive to their micro-environment, and biomaterials have the potential to alter the macrophage response and their polarization toward a regenerative rather than an inflammatory phenotype after MI. The effect of biomaterials on macrophage polarization has been investigated by several groups, focusing on the responses to cellular or acellular grafts (65), natural (66) or synthetic (67) origin and different fibers and porous sizes (68, 69). Badylak et al. compared a natural-derived scaffold made of porcine small intestinal submucosa (SIS) with its crosslinked version, known as CuffPatch (CDI-SIS) (66). The SIS patch induced a strong mononuclear response at 1-, 2-, and 4-weeks post implantation, as determined by CD163^+^ macrophages. On the other hand, the crosslinked CDI-SIS patch elicited the recruitment of an equal proportion of CD80^+^ pro-inflammatory and CD163^+^ anti-inflammatory macrophages at 1- and 2-weeks, with the anti-inflammatory population predominant in later time points and observed at the border zone rather than infiltrating the scar. Physical properties such as fiber diameter and pores size are also able to influence macrophages: larger fibers and pore size of different types of scaffolds induce the differentiation of anti-inflammatory macrophages, characterized by an increased production of wound-healing mediators such as Vascular Endothelial Growth Factor (VEGF) and basic Fibroblast Growth Factor (bFGF) and more potency in promoting capillary formation *in vitro* (68, 69). This illustrates how small changes in material properties can have significant effects on the immune response elicited by the material and how the impact of the biomaterial on the macrophage population may significantly contribute to the overall success of a biomaterial strategy.

## Hydrogel-Based Applications in Cardiac Tissue Engineering

Injectable hydrogels without addition of cells or molecular therapeutics have elicited functional improvements and prevention of left ventricular dilation in small and large animal models. However, the combination of injectable strategies with the delivery of growth factors, cytokines or other bioactive molecules shows great promise as a therapeutic approach, as hydrogels can help overcome the challenges associated with high diffusion rates and their half-life *in vivo* as discussed above (23).

### Injectable Hydrogels for Mechanical Support

Injectable hydrogels can be used as a bulking agent after MI, providing mechanical support to the infarcted heart, and preventing ventricular remodeling by attentuating wall stress (23, 70). This has not only been shown in small and large animal models but also confirmed by computational models (71–73). One of the earliest materials used for cardiac repair was fibrin, a biodegradable protein involved in the coagulation cascade (74). The field of acellular injectable materials was pioneered by Christman et al. who explored the effects of fibrin glue as post-MI bulking agent (75, 76), showing its ability to recruit endothelial cells, and to increase capillary density, probably due to the fact that fibrin contains binding domains for angiogenic factors, such as FGF (77). An ECM-derived hydrogel, specifically from pig heart ventricles, has also been investigated as an acellular bulking agent, following percutaneous delivery via trans-endocardial injection in a rat model of MI (78). Results showed a decrease in the loss of endogenous cardiomyocytes and a preserved cardiac function and were later further confirmed in pig models (79), demonstrating that providing cardiac-specific cues to the injured myocardium via decellularized cardiac ECM hydrogels is a promising strategy to reduce infarct size and promote tissue formation (23).

Hydrogels made of alginate, a naturally occurring linear polysaccharide found in brown seaweed algae, are of high interest for tissue engineering applications, due to their biocompatibility, non-thrombogenic nature and resemblance to ECM (80, 81). Alginate hydrogel injection has shown beneficial results in rat and porcine models of MI (37, 82) although, unlike fibrin, alginate must be modified with adhesive peptides to facilitate cell binding (83). Leor et al. tested the effects of intracoronary injection of alginate hydrogel in a healthy swine heart, with no evidence of biomaterial deposition, necrosis and inflammation at the injection site (82). However, being a material of natural origin, there is a risk of contaminants, including mold, yeast and bacteria, with the latter contributing to the presence of endotoxins, mitogens and pyrogens (84). Since pro-inflammatory cytokines play a role in the pathophysiology of HF (85), these contaminants must be eliminated from biomedical alginates for cardiac applications. Hence, the production of alginate hydrogels for clinical use is strictly regulated by several agencies worldwide (84). Recently, injections of a commercially available calcium alginate hydrogel (Algisyl-LVR™) were evaluated for their ability to reduce LV wall stress in HF patients with an ejection fraction (EF) <40% (72). Three months after treatment, LV wall thickness increased by 20% and end-systolic volume (ESV) decreased by 30%, showing for the first time that an acellular alginate hydrogel might be beneficial in the treatment of HF patients (72). Another study (AUGMENT-HF trial) investigated direct injections of alginate hydrogel in the myocardium of 113 patients; the material was administered through left thoracotomy with multiple intramyocardial injections and it was effective in improving cardiac function in chronic HF patients (86).

Synthetic hydrogels have also been tested as acellular *in situ* gelling systems. For example, a material consisting of α-cyclodextrin (α-CD) and poly (ethylene glycol) (MPEG-PCL-MPEG) triblock copolymer that can form a gel *in situ* has shown functional LV improvement when injected in rodents models (70, 87). Fujimoto et al. designed and tested a biodegradable thermo-responsive hydrogel made of N-isopropylacrylamide (NIPAAm), acrylic acid (AAc) and hydroxyethyl methacrylate-poly(trimethylene carbonate) (HEMAPTMC) which formed a gel at body temperature (48). Overall, this biodegradable material improved cardiac function and preserved the thickness of the ventricular wall compared with PBS injection in a rat model of MI. Specifically, the hydrogel distributed in the anterior wall and was infiltrated by macrophages and fibroblasts. This indicates that the macrophage phagocytic and secretory activity leads to a faster hydrolytic cleavage of the hydrogel *in vivo* rather than *in vitro* (48). Hydrogels are usually degraded by hydrolysis and the resulting small particles are phagocytized by macrophages at the injection site. Macrophage infiltration is associated with a higher expression of VEGF and bFGF around the newly formed vessels and these locally secreted angiogenic factors might positively influence tissue remodeling (48, 88).

## Injectable Hydrogels for the Delivery of Bioactive Factors

Due to their critical role in controlling cellular functions and orchestrating tissue regeneration, bioactive factors have received growing interest for regenerative applications in cardiovascular medicine. Many studies now highlight how the beneficial effects derived from the administration of stem cells into the infarcted myocardium are in fact due to the paracrine effects of secreted factors, rather than from direct action of the cells (89–93). This has been reviewed thoroughly elsewhere (94–96). Factors that activate specific intracellular pathways linked to cardioprotection include intermediates in the PI3K-Akt and MEK1/2-Erk1/2 pro-survival kinase pathways (97). However, as discussed above the therapeutic potential of these molecules is limited by their high rate of diffusion, short biological half-life (98), low plasma stability and low specificity to target organs (99). For example, post-MI intraperitoneal administration of a cocktail of growth factors in rats did not improve cardiac function, infarct size or neovascularization (98) and a single intracoronary infusion of FGF in patients did not significantly improve myocardial function in a phase 1 clinical trial (100). Therefore, the successful clinical translation of tissue reparative benefits of bioactive factor depends on new formulation and/or delivery approaches (99).

**Figure 2:**
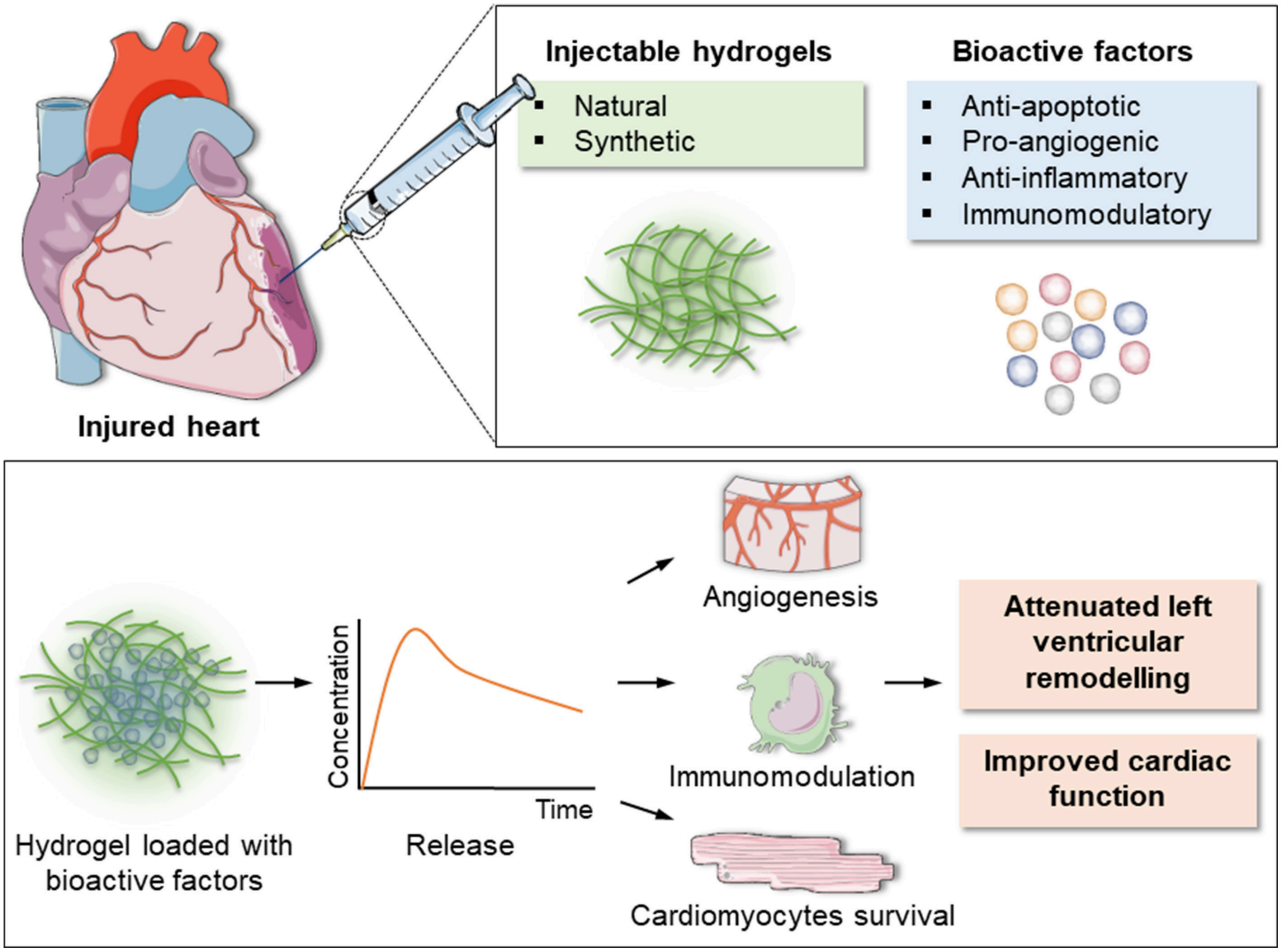
Schematic of the injectable hydrogel approach for cardiac repair. Injectable hydrogels of either natural or synthetic origin can be loaded with bioactive factors with anti-apoptotic, pro-angiogenic, anti-inflammatory, and immunomodulatory actions. The encapsulated factors are released over time, inducing formation of new blood vessels, modulation of inflammation and post-MI immune response and cardiomyocytes survival. This eventually results in reduction of left ventricular remodeling and improved overall cardiac function.

In addition to providing mechanical support, hydrogels can also serve as a water-rich matrix to encapsulate regenerative factors, representing a promising option for post-MI treatment. Bioactive factors loaded into hydrogels to test their ability to locally modulate cardiac repair include angiogenic (VEGF, FGF), anti-apoptotic (IGF-1, NRG-β), proliferative (SDF-1) and immunomodulatory (IL-10, TIMP-3) molecules (Figure 2).

## Angiogenic Factors

Angiogenesis refers to the development of blood vessels from a pre-existing vascular bed. Clinically, the objectives can be to either block vessel formation to treat tumors or to stimulate angiogenesis in states of insufficient blood flow such as the ischemic heart disease (101). In the context of myocardial infarction, angiogenic therapy may salvage the ischemic tissue, especially in the early stages post-MI. Angiogenesis occurs in the granulation tissue during the proliferative phase post-MI. However, supporting neovascularization of the viable, surrounding myocardium at the infarct border zone may improve the process of tissue remodeling (102). Most studies have focused on the regenerative potential of VEGF and FGF. Localized and low-dose delivery of pro-angiogenic factors is particularly important because, regardless of how efficient the uptake, a considerable proportion of an angiogenic factor injected into a vessel supplying the target tissue will spill into the systemic circulation and expose non-target tissue to its biologic effects (103, 104). This is highly relevant for VEGF and its angiogenic effects because high doses of VEGF may result in the unregulated formation of haemangiomas (105) or vascular leakage that leads to oedema and nitric oxide-dependent hypotension (106, 107). Thrombocytopenia and renal toxicity are the most likely side effects of high doses of FGF (108). Additionally, the immune system is not normally exposed to high doses of these factors and can potentially develop antibodies against them, leading to decreased administration efficacy. Hence, the use of injectable hydrogels that allow a sustained yet localized delivery of pro-angiogenic factors is a promising option for post-MI treatment. Examples of injectable biomaterials tested for the delivery of pro-angiogenic factors in animal models are summarized in Table 1.

**Table 1:** Exemplar injectable biomaterials used for the *in vivo* delivery of pro-angiogenic growth factors in animal models of MI.

**Biomaterial**	**Growth factor**	**Animal model**	**Time of administration**	**Outcome**	**References**
PEG-Fibrinogen	VEGF	Rat MI	Immediately after MI	Increased arterial density and improved cardiac function	(109)
Thermo-responsive aliphatic polyester	VEGF	Rat MI	1 week post MI	Improved angiogenesis and cardiac function with smaller infarcts	(52)
Acidic gelatin hydrogel microspheres	bFGF	Canine MI	2 weeks post MI	Improved collateral circulation near the coronary occlusion	(110)
Acidic gelatin hydrogel microspheres	bFGF	Rat chronic MI	4 weeks post MI	Improved fractional shortening and neovascularization. Increased collagen III/I ratio in the fibrotic scar.	(111)
Acidic gelatin hydrogel microspheres	bFGF	Canine chronic MI	4 weeks post MI	Improved fractional shortening and capillary density	(112)
Thermo-responsive chitosan	bFGF	Rat MI	1 week post MI	Improved arteriogenesis, ventricular remodeling, and cardiac function	(113)
Decellularised pericardial ECM	bFGF	Rat MI	1 week post MI	Enhanced vascularization with newly formed vasculature anastomosed with existing vessels	(114)
Dex-PCL-HEMA/PNIPAAm	bFGF	Rat MI	Immediately after MI	Reduced collagen, neoangiogenesis, and improved cardiac function	(115)
(p[NIPAAm-co-PAA-co-BA])	bFGF	Rat MI	Immediately after MI	Increased capillaries density. Improved cardiac function with increased thickness of myocardial wall	(116)

### Vascular Endothelial Growth Factor (VEGF)

VEGFs are a family of glycoproteins of which VEGF-A (also called VEGF-1) is the most extensively studied. Other members of the family, which shares structural homology, are VEGF-C, VEGF-B, VEGF-D and VEGF-E (117). Four species of VEGF-A have been identified, differing in the number of amino acids (VEGF_121_, VEGF_165_, VEGF_189_, and VEGF_206_). VEGF_165_ is the most abundant and predominant isoform of VEGF in the heart. Since it is the major regulator of angiogenesis in the heart, VEGF_165_ is also the most commonly used for therapeutic angiogenesis post MI (117). The first study showing its cardioprotective effects dates back to 1997 (118). Since then, many approaches have been tested for VEGF delivery to the ischemic heart, including gene therapy (119–121) and intravenous and intracoronary administration (122, 123). The clinical trial VIVA assessed the effects of intracoronary infusion of recombinant human vascular endothelial growth factor (rhVEGF) in patients with chronic myocardial ischemia, showing a well-tolerated safety profile but no clinical improvements over placebo by day 60 (124). However, by day 120, rhVEGF at high dose led to significant improvement in angina and increasing trends in the Exercise Treadmill Test (ETT). As in several other trials (122, 125, 126), VIVA showed a dose-dependent effect, highlighting once more the need for controlled and sustained release.

Various types of hydrogels, either of natural or synthetic origin, have been used for the delivery of VEGF. PEG-based hydrogels provide several advantages for *in vivo* applications and they have been extensively used in regenerative medicine due to their high water content and the fact that they can provide a three-dimensional environment similar to soft tissue, allowing distribution of oxygen, nutrients and metabolites (127–129). Recently, a biosynthetic injectable hydrogel consisting of polyethene glycol and fibrinogen (PEG-fibrinogen) loaded with VEGF and administered by intramyocardial injection showed myocardial function protection and improved vascularization in a rat MI model (109).

The most common approach for the encapsulation of a bioactive factors into an injectable hydrogel is simply by mixing it with the polymer solution. However, previous studies have shown that if angiogenic factors are immobilized on a scaffold, their angiogenic potential is enhanced (130, 131). Along these lines, a slightly different approach undertaken by Wu et al. was to conjugate VEGF to a [Poly (δ-valerolactone)-*block*-poly (ethylene glycol)-*block*-poly (δ-valerolactone) (PVL-*b*-PEG-*b*-PVL)] instead of mixing it with the solution (52). All the conditions (hydrogel alone, hydrogel mixed with VEGF and hydrogel conjugated with VEGF) were able to significantly attenuate adverse cardiac remodeling. However, VEGF-conjugated hydrogels were better in boosting angiogenesis, likely because the conjugation was able to extend the biological activity of VEGF over the 42 days of biomaterial degradation (52).

These studies were mainly focused on material design and characterization, and cardiac function was assessed by echocardiography. Immunomodulatory effects of VEGF were not tested. Importantly though, VEGF family factors not only have a direct effect on endothelial cell survival and proliferation, thus control new vessel formation and permeability, but also recruit inflammatory and regenerative cells such as myeloid and progenitor cells (102).

### Basic Fibroblast Growth Factor (bFGF)

bFGF, a 16 kDa monomeric factor, is the most potent angiogenic factor in the FGF family and it affects migration and proliferation of endothelial cells, smooth muscle cells and fibroblasts (115). However, its short half-life of only 3 min and side effects induced by high dose administration have hindered a robust clinical translation so far. Since it was first demonstrated that FGF could increase the number of capillaries and arterioles in the infarcted dog heart (132), it has been widely used in animal models (47, 104) and clinical trials (100, 133, 134), showing treatment safety but not yet demonstrating efficacy.

The first biomaterials used for local delivery were bFGF-impregnated acidic gelatin hydrogel microspheres (AGHM), improving collateral circulation to the infarcted area after coronary occlusion in dogs significantly more than the free-form bFGF (110). Gelatin microspheres were also used to deliver bFGF in several hindlimb ischemia large animal models (135, 136). These studies showed promising results, leading to progression to clinical trials in patients with critical limb ischemia (137, 138). Currently, the same approach is being tested in small (111) and large animal models (112) of chronic MI. The improved cardiac contractile function seen in these recent studies shows how treatment with the sustained release of bFGF from gelatin hydrogels could be clinically translated not only for peripheral cardiovascular diseases but also for chronic MI.

In addition to gelatin microspheres, temperature-responsive chitosan was used for the delivery of FGF post-MI by Wang et al. (113). In this study, FGF was encapsulated in a thermo-responsive chitosan hydrogel upon intra-myocardial injection. This system significantly improved cardiac function compared to injecting FGF alone in a rat model of chronic MI (hydrogel injection 1-week post-MI) (113). bFGF also retains its bioactivity when delivered through natural-derived hydrogels such as decellularized pericardial ECM (114) or synthetic materials such as (*p*[NIPAAm-co-PAA-co-BA]) (50). Recently, bFGF (139) has shown overall amelioration of ischemic injury caused by MI in a mouse model using coacervate hydrogels (60).

As an example of the immunomodulatory responses to hydrogels, Garbern et al. designed a dual responsive polymer made of poly(*N*-isopropylacrylamide-co-propyl acrylic acid-co-butyl acrylate) (*p*[NIPAAm-co-PAA-co-BA]) which is a liquid at pH 7.4 and 37°C and forms a gel at pH 6.8 and 37°C. They hypothesized that the ability of this polymer to form a reversible gel under the acidic conditions of the ischemic myocardium (pH 6.8) would allow it to first act as a depot system for the release of bFGF, and secondly to promote polymer dissolution once the tissue has returned to physiological pH (116). The system was tested for its efficacy *in vivo* in a rat model of MI, showing that it was able to provide a spatiotemporally controlled release of bFGF which in turn promoted angiogenesis, and improved cardiac function (116). Interestingly, the inflammatory response quantified by CD45 staining at day 28 was higher in animals injected with the polymer compared to saline. Near the polymer injection site, there was a significant macrophage infiltration and foreign body giant cells were also found, evidencing a chronic inflammation response. Macrophages promote angiogenesis and produce proangiogenic factors such as VEGF, IGF-1, and bFGF (140). Hence, it is possible that the enhanced presence of macrophages in polymer-treated animals has a beneficial effect by further improving the angiogenic response. However, time points past 28 days are needed to elucidate if the inflammatory response will be resolved appropriately to avoid detrimental effects long-term.

## Anti-Apoptotic Factors

Cardiomyocytes apoptosis is responsible for most of the myocyte death from the early stages of MI to the progression to HF. It is in fact not only detected in the infarct area, but it extends to the viable myocardium in remote non-infarcted regions, characterizing all post-MI phases (61, 141, 142). Anti-apoptotic therapeutic interventions therefore seem a potential therapeutic strategy and could involve the use of injectable hydrogels to deliver anti-apoptotic molecules to attenuate the loss of viable myocardium. Insulin-like Growth Factor 1 (IGF-1) and Hepatocyte Growth Factor (HGF) can both activate the PI3K/Akt pathway, enhancing cell survival, and reducing cardiomyocyte apoptosis resulting in improved cardiac function (61, 143). Examples of chemokines able to prevent myocyte apoptosis with demonstrated effectiveness are Granulocyte colony-stimulating factor (G-SCF) (144) and erythropoietin (EPO) (145, 146).

### Insulin-like Growth Factor-1 (IGF-1)

IGF-1 is a 72 kDa polypeptide which plays key roles in cell survival, proliferation, and differentiation via different signaling pathways such as Ras-Raf mitogen active protein kinases and phosphoinositide 3-kinase/Akt pathway (147). Buerke et al. carried out the first study to show acute cardioprotective effects of IGF-1, demonstrating that it reduced myocardial necrosis, apoptosis, and neutrophil accumulation (148). Since then, the beneficial effects of IGF-1 post-MI have been investigated by several groups employing several strategies including IGF-1 myocardial overexpression (149, 150), gene therapy (151), and systemic (152) and local administration (153). IGF-1 transgenic overexpression not only activates survival signaling pathways in the cardiomyocytes, but also mediates myocardial repair by modulating the inflammatory response post-MI with decreased expression of the pro-inflammatory cytokines IL-1β and IL-6 and increased expression of the anti-inflammatory IL-4 and IL-10 compared to wild type mice (154). Moreover, cardiac-specific overexpression of IGF-1 resulted in early accumulation of innate immune cells at day 3 post-MI, with a reduction of inflammatory myeloid populations (149). Similar trends were found when IGF-1 was delivered through a single intravenous injection of AAV9 containing a cardiac-restricted IGF-1 isoform (151). These findings are supported by previous studies that identified anti-inflammatory effects of IGF-1 in hyper-inflammatory conditions, which were due to the induction of regulatory T cells (155, 156).

Addition of IGF-1 to cell therapy strategies enhances the benefit of cell transplantation by promoting cell survival (157). Recently, IGF-1 was delivered together with Bone Marrow Stem Cells (BMSC) in a rabbit model of MI through biotinylated self-assembly peptides and was able to suppress cardiomyocyte apoptosis and promote the expression of cardiomyocyte-specific proteins (158). These findings were confirmed for different regenerative medicine applications such as cartilage repair (159), peripheral vascular diseases (160) or acute kidney injury (161). IGF-1 release with maintained bioactivity was shown *in vitro* using an injectable, thermo-sensitive hydrogel composite capable of gelling within 6 s (162). In this study, IGF-1 was also able to increase the survival of mesenchymal stem cells (MSCs) encapsulated in the gel, making the system an attractive strategy for cardiac tissue engineering. Collectively these studies provide evidence of how the combination of IGF-1 with therapeutic stem cells delivery is a promising approach to increase the survival and consequently the engraftment of transplanted cells. To date, there are no studies using injectable biomaterials to deliver IGF-1 as a single factor to the heart *in vivo*, but several reports of combined delivery which will be covered in the next section.

### Neuregulin-1β

Neuregulin-1β (NRG-1β), a member of the epidermal growth factor (EGF) family, is another antiapoptotic factor that has recently gained attention as a therapy for cardiovascular diseases. The critical role of NRG-1β in both cardiac development and maintenance of normal adult heart function is well-established (163). NRG-1β receptor is expressed by human cardiac ventricular fibroblasts and NRG-1β treatment of these cells under stress has a pro-survival action (164). Moreover, activation of the NRG-1β pathway induces the production of angiopoietin-2 (Ang-2) and brain-derived neurotrophic factor (BDNF), which have pro-angiogenic and pro-survival effects, respectively (164). Systemic administration of NRG has demonstrated efficacy in reducing fibrosis and improving LV function in cardiomyopathy animal models (165–167), leading to clinical trials employing daily infusion of high dose recombinant NRG, which showed a modest improvement in LV ejection fraction in comparison to placebo or low dose administration (168). However, this approach involves daily infusions and off-target exposure and therefore, novel clinically translatable strategies are being investigated. As an example, Cohen et al. developed a hydroxyethyl methacrylate (HEMA)-based injectable hydrogel system to directly deliver NRG to the myocardial border zone in a rat MI model, and showed augmented cardiomyocyte mitotic activity, decreased apoptosis, and greatly enhanced LV function without off-target exposure (169). Other injectable systems that have been tested for NRG delivery include PLGA-microparticles, which showed increased ejection fraction and also improved angiogenesis when delivered with a percutaneous intramyocardial injection in rat (170) and porcine preclinical models of MI (171).

## Immunomodulatory Bioactive Molecules

Due to the role of excessive inflammation in exacerbating myocardial damage post-MI, a range of immunomodulatory strategies have been attempted in clinical as well as in experimental studies (55). Although current standard pharmacotherapy post-MI has potent immunomodulatory functions (172). systemic administration of these agents has so far shown little benefit, and adverse effects of systemic immunomodulation limit their clinical translation.

Local delivery using hydrogels may be a promising alternate strategy. Some naturally derived materials be intrinsically anti-inflammatory. For example, chitosan scavenges Reactive Oxygen Species (ROS) *in vitro* and *in vivo*, which could explain the improved cardiac function following chitosan hydrogel injection post-MI (173). High molecular weight Hyaluronic Acid (HA) is another material of natural origin with ROS-scavenging properties (174, 175). Recently, also a fully synthetic hydrogel made of polyglycerol sulfate-based PEG showed intrinsic anti-inflammatory actions when tested in an *in vitro* model of osteoarthritis (176). However, for most biomaterials, either of natural or synthetic origin, loading of anti-inflammatory therapeutics is necessary to modulate the inflammatory cardiac microenvironment.

Anti-inflammatory cytokines share the same challenges for *in vivo* delivery as other bioactive factors, most prominently a short half-life. IL-10 is a pleiotropic cytokine with broad immunoregulatory and anti-inflammatory activities (177). Daily subcutaneous injection of IL-10 in a rat MI model resulted in significantly decreased expression of pro-inflammatory cytokines and reduced macrophages infiltration (178). Its half-life of only 2.7–4.5 h (179), however, means that high doses and repeated injections are needed, leading to increased risk of side effects and high treatment cost. To overcome these issues, an injectable coacervate hydrogel was recently implemented for the delivery of IL-10 combined with bFGF in a mouse model of acute MI (180). A single coacervate treatment of 500 ng each of bFGF and IL-10 led to long-term synergistic benefit post-MI with ameliorated LV contractile function and LV dilation. IL-2 and IL-19 have also proven beneficial against post-MI remodeling when delivered exogenously (181), (182). IL-2 is important for the survival of regulatory T cells, whereas IL-19 inhibits proinflammatory macrophages, making them promising candidates for incorporation into injectable biomaterial systems.

Besides the delivery of exogenous anti-inflammatory factors, inhibition of endogenous pro-inflammatory molecules may also achieve immunomodulation. Tumor necrosis factor (TNF)-α antagonism ameliorates ischemia/reperfusion injury (183) and hydrogels delivering anti-TNF-α have been used for several applications such as burns (184), wound healing (185) or inflammatory bowel disease (IBD) (186). Therefore, a therapeutic strategy using the same approach to salvage myocardial tissue post-MI could be promising.

An emerging and translationally relevant therapeutic approach to mitigate post-MI inflammation and remodeling involves the localized augmentation of Tissue Inhibitor of Metalloproteinases-3 (TIMP-3), a negative regulator of matrix metalloproteinase (MMP) activity. The cause-effect relationship between MMP induction and adverse LV remodeling has been established through pharmacological MMP inhibition (187). However, translation of systemic administration of MMP inhibitors has encountered concerns around potential off-target side effects when the delivery is not localized (188). Injectable hydrogels represent an attractive alternate means of delivery for TIMP-3. After the first proof of concept showing that sustained regional delivery of TIMP-3 through a degradable hyaluronic acid hydrogel can effectively block adverse LV remodeling (189, 190), the hydrogel was recently modified with an MMP-cleavable peptide (51). TIMP-3 release by this system was tested in pigs following coronary artery ligation, with promising results showing improved cardiac geometry and function (51). In all of the above studies, the favorable effects of TIMP-3 on post-MI remodeling are associated with an attenuation of the local inflammatory process. TNF and TNF receptor mRNA were increased in all post-MI groups but were lower when delivering TIMP-3 in comparison to saline injection. The same trend was shown for markers of macrophages maturation and infiltration such as monocyte chemoattractant protein-1 (MCP-1) and macrophage inflammatory protein-1α (MIP-1α). Moreover, TIMP-3 release increased myofibroblast density, an index of a stronger and more flexible scar (191). This supports the notion that TIMP-3 improves LV geometry through modifying biological mediators of the adverse post-MI remodeling process (189).

Examples of anti-apoptotic and anti-inflammatory factors delivered in animal models are summarized in Table 2.

**Table 2:** Exemplar injectable biomaterials used for the *in vivo* delivery of anti-apoptotic and anti-inflammatory bioactive molecules in animal models of MI.

**Biomaterial**	**Growth factor**	**Induced mechanism**	**Animal model**	**Time of administration**	**Outcome**	**References**
Hydroxyethyl methacrylate (HEMA)	Neuregulin-1β	Anti-apoptosis	Rat MI	Immediately after MI	Augmented cardiomyocytes mitotic activity and decreased apoptosis. Improved cardiac function with reduced left ventricular dilation	(169)
Poly(lactic-co-glycolic acid) microparticles	Neuregulin-1β	Anti-apoptosis	Swine ischemia/ reperfusion	1 week post MI	Improvement in systolic and diastolic cardiac function and decrease in transmural infarct progression	(171)
Hyaluronic acid hydrogel	TIMP-3	Anti-inflammatory	Swine MI	Immediately after MI	Improved LV ejection fraction and reduced LV dilation. Marked reduction in pro-inflammatory cytokines	(189)
Metalloproteinase-responsive hyaluronic acid hydrogel	TIMP-3	Anti-inflammatory	Swine MI	Immediately after MI	Reduced LV dilation and wall thinning. Decrease in transcriptional profile for pro-fibrotic pathways	(51)

## Combined Delivery of Multiple Growth Factors

It is likely that a synergistic approach with simultaneous administration of multiple factors may more accurately mimic their natural mode of action and show more robust beneficial effects. Further, incorporation of multiple agents in an optimized ratio may allow for spatiotemporally controlled sequential delivery of several bioactive factors with synergistic effects.

The PEG-fibrinogen hydrogel described above (109) was also used for a dual delivery of VEGF and angiopoietin-1 (Ang-1), another growth factor known to induce angiogenesis and maturation of newly formed blood vessels both *in vitro* (192) and *in vivo* (193, 194). The study demonstrated significant improvement in cardiac function at 4 weeks in rats treated with this combination of factors (195).

VEGF was also combined with HGF in a bioactive hydrogel comprising PEG linked to a protease-degradable peptide to take advantage of the high levels of proteases found in the ischemic myocardium (196). This stimulus-responsive system triggers the release of the encapsulated factors when remodeling occurs, and it was tested in a rat model of MI (197). Interestingly, when cardiac function was measured at day 7 post MI, only the empty hydrogel showed a significant improvement in function, as measured by fractional shortening. Conversely, at day 21 there was a significant improvement in function only in the group that received the hydrogel with the combination of factors (197). This suggests that VEGF and HGF are not released fast enough to have an effect in the acute phases, but they were efficient in inhibiting fibrosis and inducing angiogenesis at later time points and only if administered together.

Another common approach is to couple VEGF with other factors such as platelet-derived growth factors (PDGF-BB) that support the stability and the connectivity of new vessels by recruiting smooth muscle cells (198). PDGF signaling plays an essential role in cardiovascular development (199) and in both mouse and humans the PDGF family consists of four ligands, PGDF-A-D. However, only PDGF-A and PDGF-B are capable of forming functional heterodimers (199) and have been shown to protect cardiomyocytes from apoptosis and improving contractile function of an engineered heart tissue (200). VEGF_165_ and PDGF-BB have been co-delivered through an alginate hydrogel, leading to improvements in cardiac function more than with each factor separately (198). Moreover, endothelial cells promote cardiomyocytes survival via PDGF signaling (201). A recent proangiogenic combination strategy involved the delivery of Stromal Derived Factor-1 (SDF-1) and an angiogenic small tetrapeptide (Ac-SDKP) for bone marrow stem cell recruitment and angiogenesis, respectively (36). It showed how dual therapeutic factors can provide an injectable 3D microenvironment for recruiting MSCs into the ischemic area and, at the same time, play a role for stimulating neoangiogenesis.

IGF-1 has been coupled with several other factors for dual delivery and tested *in vivo* in MI models. Sequential delivery of regenerative factors is thought to be more effective than simultaneous delivery because it mimics the naturally-occurring healing phases (47). Alginate hydrogels were used for the sequential delivery of IGF-1 and HGF (202) in a rat model of MI. To increase the local potency of the factors at the infarct exploiting a localized stimulus, the delivery was carried out in a partially-crosslinked alginate solution, previously shown to undergo gelation in response to the high concentration of calcium ions that characterize the ischemic myocardium. The alginate hydrogel formed *in situ*, creating a local reservoir for the factors and providing an additional barrier against protein diffusion (202). The sequential release was achieved by varying the initial loading concentrations of the two factors. The study showed attenuated infarct expansion and diminished fibrosis, together with enhanced angiogenesis at the infarct site following dual delivery.

Furthermore, dual delivery of IGF-1 and VEGF was implemented in a study using injectable gelatin microspheres in a rat MI model (203). This showed how the neoangiogenesis promoted by VEGF can potentiate the anti-apoptotic actions of IGF-1, resulting in a marked reduction of infarct size associated with improved cardiac function. Nelson et al. encapsulated both IGF-1 and bFGF in a thermo-responsive synthetic hydrogel made of Poly(NIPAAm-co-HEMA-coMAPLA) (47) and previously described (204). Interestingly, cardiac function in the hydrogel-treated animals was improved at the 16 weeks timepoint compared to saline injection. However, both functional and histological evaluation showed no further benefit with the encapsulation of the factors compared to the empty gel (47). This could be due to the late time point chosen for gel injection in this study (2 weeks post-MI), since IGF-1 is more effective in preventing apoptosis in the early phases after an infarction (205). The material used in this study had a slow *in vivo* degradation rate (4–5 month); while the gold-standard for *in situ* degradation timing is still debated, a material designed to provide mechanical support should have a degradation time longer than 2 months (47, 206). The improvement shown in this study, regardless of factors incorporation, could then be explained by the long degradation rate, which allowed a slow shift of load-bearing responsibilities to the newly formed tissue. Moreover, a slow-degrading material remains in the tissue for enough time to support cell recruitment at the injection site, contributing to the overall success of the approach. Notably, macrophage infiltration to the site of injury was still significant 16 weeks after hydrogel injection (47). The majority of these cells stained positive for CD163, a scavenger receptor specifically expressed on the surface of activated anti-inflammatory macrophages and monocytes (207).

A combination of IGF-1 and bFGF increased ejection fraction and reduced pathological hypertrophy when delivered through a pH-switchable hydrogel ^[49]^ in a porcine model of chronic MI with catheter-based state of the art technology (208). These results are particularly promising not only for the use of a highly translatable delivery system in a large animal model but also due to the time of injection (4 weeks post-MI) which has clinical relevance for chronic MI in patients. A synergistic effect of stromal-derived factor-1 (SDF-1) and the small angiogenic tetra peptide Ac-SDKP was demonstrated in a rat chronic MI model using an injectable biomimetic hyaluronic acid hydrogel for the dual delivery to the heart (36). SDF-1 increases cardiomyocyte survival in the infarct zone and promotes stem cells mobilization and stabilization (209, 210). Examples of dual delivery approaches tested *in vivo* in animal models are summarized in Table 3. Recently, Awada et al. demonstrated that proper spatial and temporal cues from proteins are essential by using for the first time a combination of three complementary factors, TIMP-3, bFGF, and SDF-1α embedded in heparin-based coacervates for sustained release regulated in a timely fashion (211). TIMP-3 reduced ECM degradation early after MI, while bFGF and SDF-1α triggered a robust angiogenic process and progenitor cell recruitment over an 8 weeks period (211). A recent trend involves the delivery of a cocktail of stem cell-derived bioactive molecules known as secretome, which includes cytokines, growth factors, and exosomes. A nanocomposite injectable hydrogel loaded with secretome from human adipose-derived stem cell has been tested for its regenerative potential *in vitro* and *in vivo* in an acute MI rat model (212). The injection of the secretome-loaded hydrogel in the peri-infarct area provided cardioprotection promoting increased angiogenesis and reduction of cardiac remodeling (212). In summary, a successful outcome of this approach is dependent upon the choice of the right growth factors, in the right combination and at the right concentration.

**Table 3:** Exemplar injectable biomaterials used for the *in vivo* combined delivery of multiple bioactive factors in animal models of MI.

**Biomaterial**	**Growth factor**	**Induced mechanism**	**Animal model**	**Time of administration**	**Outcome**	**References**
PEG-fibrinogen hydrogel	VEGF + Ang-1	Pro-angiogenesis + stabilization of newly formed vessels	Rat MI	Immediately after MI	Improvement in EF and neoangiogenesis, more significant with dual delivery compared to single factor delivery.	(195)
Protease-responsive PEG-based hydrogel	VEGF + HGF	Pro-angiogenesis	Rat ischemia/ reperfusion	Immediately after injury	Significant increase in angiogenesis, stem cell recruitment, inhibition of collagen deposition and decrease in fibrosis with dual delivery.	(197)
Alginate hydrogel	VEGF + PDGF	Pro-angiogenesis + recruitment of smooth muscle cells to support new vessels	Rat MI	Immediately after MI	Higher density of mature vessels and improvement in cardiac function.	(198)
Biomimetic hyaluronic acid hydrogel	SDF-1 + Ac-SDKP	Pro-angiogenic + bone marrow stem cell recruitment	Rat MI	Immediately after MI	Improved LV function, increased angiogenesis, and wall thickness.	(36)
Affinity-binding alginate microbeads	IGF-1 + HGF	Anti-apoptosis + pro-angiogenesis	Rat MI	Immediately after MI	Attenuation of infarct expansion and reduced scar fibrosis.	(202)
Gelatin hydrogel microspheres	IGF-1 + VEGF	Anti-apoptosis + pro-angiogenesis	Rat MI	Immediately after MI	Decreased apoptosis and inflammation. Significant neoangiogenesis. Marked reduction of infarct size and improved cardiac function.	(203)
Thermo-responsive Poly(NIPAAm-co-HEMA-coMAPLA) hydrogel	IGF-1 + bFGF	Anti-apoptosis + pro-angiogenesis	Rat MI	2 weeks after MI	Improvement in cardiac function with empty gel. No added benefit of GF addition.	(47)
pH-switchable supramolecular UPy hydrogel	IGF-1 + bFGF	Anti-apoptosis + pro-angiogenesis	Porcine chronic MI	4 weeks post MI	Reduced pathological hypertrophy and increased capillarization.	(208)
PLGA and PEG-PLGA microparticles	NRG-1β + bFGF	Anti-apoptosis + pro-angiogenesis	Rat MI	4 days post MI	Enhanced EF and neoangiogenesis. No difference between PLGA and PEG-PLGA system.	(170)
Heparin-based coacervate hydrogel	IL-10 + bFGF	Anti-inflammatory + pro-angiogenesis	Mouse MI	Immediately after MI	Improvement in long-term LV contractile function and ameliorated LV dilation. Enhanced revascularization of the infarcted area.	(180)
Fibrin coacervate gel	TIMP-3 + bFGF + SDF-1α	Anti-inflammatory + pro-angiogenic + progenitor cells recruitment	Rat MI	Immediately after MI	Reduced ventricular dilation, inflammation and ECM degradation. Improved cardiac function.	(211)

A different strategy involves the use of injectable hydrogel for the delivery of RNAi molecules such as siRNA, miRNA, and shRNA to boost regeneration. RNAi molecules have a short half-life and are rapidly cleared if delivered systemically (213). Therefore, as bioactive factors, they could highly benefit from a localized and controlled delivery, as reviewed in Wang and Burdick (213). Although the field is still in its infancy, some recent applications for cardiac repair have already shown promising results (214–216).

## Challenges and Clinical Perspectives

The physical and biological properties of the injected material, together with timing and location of injections and distribution of the hydrogel in the cardiac wall are some of the parameters that may contribute to a successful post-MI treatment.

A consensus should be reached on what is considered a successful outcome for an injectable biomaterial study and what is the best parameter, or parameter combination, to focus on. In the studies presented in this review, wall thickness, fractional shortening, LV volumes, ejection fraction, infarct size, and vascularization of the infarct have all been considered. In the clinical setting, End Systolic Volume (ESV) has been shown to be the best predictor of survival and re-hospitalization (217). Specific considerations for myocardial applications are:

### Immunomodulatory Aspects

Timing and location are crucial for the control of inflammation in the damaged heart. As we gain a more detailed understanding of the shifting post-MI immune response, an equally dynamic therapeutic protocol would engage a spectrum of immunomodulatory interventions during the post-MI period. Targeted delivery of these therapeutic agents is equally critical: although clinical trials involving immunomodulation use broad immunosuppressive protocols that have proven partially successful, a more localized dispatch of commonly used immunosuppressive treatments in the heart may greatly improve their immunomodulatory efficacy while minimizing systemic adverse effects. While production of engineered materials has historically been focused on maximizing their bioinert properties, recent studies reflect a shift toward active biomaterials that elicit a controlled inflammatory response as described above. Although several considerations are necessary when designing an injectable hydrogel, alginate hydrogels have already been used in clinical settings (218). However, one disadvantage of these materials is that they might retain some surface antigens capable to elicit an immune response. Conversely, synthetic materials are designed with defined chemical compositions that does not elicit a foreign body response, but often require functionalization with appropriate bioactive molecules to support cell attachment and survival. In the future, hydrogels with natural-synthetic hybrid compositions may allow more precise delivery of immunomodulatory biologics at the right place and time.

### Effects on the Cardiac Conduction System

A significant concern when injecting a hydrogel into the myocardial wall is its possible effect on the cardiac conduction system. This is particularly important for patients eligible for a biomaterial therapy who may already have an increased risk of developing ventricular arrhythmias (219). Suarez et al. used optical mapping to study the effect of interstitial spread with PEG-based hydrogels in rats and did not observe changes to action potential propagation with high spreading materials characterized by slow gelation times (219). Conversely, materials with a quicker gelation time that form a bolus could potentially be a substrate for arrhythmias (219). This interesting study about a material design criterion that is often overlooked needs to be repeated in large animal models and with other biomaterials to provide better foundations for safer clinical use of injectable hydrogels.

### Biodegradability

Degradation time of a hydrogel is a key parameter but data about biodegradability *in vivo* are still lacking. For myocardial tissue engineering, a material is considered biodegradable if degradation occurs through hydrolytic or enzymatic activity *in vivo* and if the degradation products comply with the requirements of both biodegradability and biocompatibility (29). In general, a material should persist long enough to have the desired effect but not longer than necessary to affect the repair process. Therefore, biomaterials designed for delivery of bioactive factors should persist *in vivo* for at least 1 week since most of the cell death occurs within the first few days after MI and should be fully degraded in 6–8 weeks. Materials of natural origin such as ECM-derived materials provide the correct composition to allow cell adhesion and survival and are degraded within days to weeks by enzymes produced by the cells into biodegradable and biocompatible products. Given the extensive research conducted in small animal models, the next step for a better *in vivo* characterization of biodegradability will require studies in large animal models, and longer-term follow-up to delineate the mechanisms in which these materials act, both biologically and mechanically.

## Conclusions

Numerous preclinical studies on hydrogel-mediated delivery of bioactive factors to the heart have shown therapeutic benefit in terms of global cardiac parameters such as improved or maintained left ventricular geometry and ejection fraction. It is now widely acknowledged that the mechanism of action of successful cell therapy for cardiac repair occurs via paracrine signaling (220). Amplifying this therapeutic approach by local hydrogel-mediated delivery of salutary factors to improve cell survival, vascularisation and prevent excessive inflammation represents a promising avenue that could accelerate the development of novel treatment for MI and HF. The field of controlled-release systems through biomaterials is still in its infancy but offers significant potential to modulate immune response, modulate inflammation, and minimize infarct scar, promote angiogenesis to prevent cell death, and provide the necessary extracellular milieu for constructive cardiac remodeling after injury.

## Author Contributions

AF conceived and drafted the manuscript. SS contributed to the writing and the editing. SS, MS, and NR critically revised the work. All authors were responsible for the final approval of the completed version.

### Conflict of Interest Statement

The authors declare that the research was conducted in the absence of any commercial or financial relationships that could be construed as a potential conflict of interest.
